# The Genetic Diversity and Geographic Differentiation of the Wild Soybean in Northeast China Based on Nuclear Microsatellite Variation

**DOI:** 10.1155/2018/8561458

**Published:** 2018-06-06

**Authors:** Hongkun Zhao, Yumin Wang, Fu Xing, Xiaodong Liu, Cuiping Yuan, Guangxun Qi, Jixun Guo, Yingshan Dong

**Affiliations:** ^1^Key Laboratory of Vegetation Ecology, Ministry of Education, Institute of Grassland Sciences, Northeast Normal University, Changchun 130024, China; ^2^Institute of Crop Germplasms, Jilin Academy of Agricultural Sciences, Gongzhuling 136100, China; ^3^Institute of Soybean Research, Jilin Academy of Agricultural Sciences, Changchun 130033, China

## Abstract

In this study, the genetic diversity and population structure of 205 wild soybean core collections in Northeast China from nine latitude populations and nine longitude populations were evaluated using SSR markers. A total of 973 alleles were detected by 43 SSR loci, and the average number of alleles per locus was 22.628. The mean Shannon information index (*I*) and the mean expected heterozygosity were 2.528 and 0.879, respectively. At the population level, the regions of 42°N and 124°E had the highest genetic diversity among all latitudes and longitudes. The greater the difference in latitude was, the greater the genetic distance was, whereas a similar trend was not found in longitude populations. Three main clusters (1N, <41°N-42°N; 2N, 43°N-44°N; and 3N, 45°N–>49°N) were assigned to populations. AMOVA analysis showed that the genetic differentiation among latitude and longitude populations was 0.088 and 0.058, respectively, and the majority of genetic variation occurred within populations. The Mantel test revealed that genetic distance was significantly correlated with geographical distance (*r* = 0.207, *p* < 0.05). Furthermore, spatial autocorrelation analysis showed that there was a spatial structure (*ω* = 119.58, *p* < 0.01) and the correlation coefficient (*r*) decreased as distance increased within a radius of 250 km.

## 1. Introduction

The annual wild soybean (*Glycine soja* Sieb. and Zucc.), the direct progenitor of the cultivated soybean (*Glycine max* (Linn.) Merr.), is a predominantly self-pollinated annual plant species [1, 2]. It is widely distributed across most provinces of China, with the exceptions of Qinghai, Xinjiang, and Hainan [3]. In Northeast China, the wild soybean is well known for its abundant populations, high population density, and rich phenotypic types [4]. A total of 48 wild soybean in situ reserves have been established in China, 14 of which are located in Northeast China. A total of 8518 wild soybean accessions have been ex situ conserved in the National Gene Bank, and nearly half were collected from Northeast China [5]. Some reports have suggested that Northeast China was probably a very important diversity center for wild soybeans [6–8].

Genetic diversity is essential to population stability and is the basis of the evolution of species [9]. The genetic diversity and population structure of wild soybeans have been described in many reports [10–13]. The wild soybean in Northeast China, which is a very important ecotype, has been used in many previous studies [14–18]. The genetic diversity of wild soybeans in Northeast China was often compared with that in other areas using morphological traits and various molecular markers. Alternatively, wild soybeans from some specific areas have been analyzed for their distribution patterns, origin, evolution, classification, and so on. Accordingly, results have not been consistent because different accessions (or populations), numbers of samples, and analysis methods have been used. SSR markers have proven to be a reliable tool for determining the diversity of the wild soybean [19]. However, regarding wild soybeans in Northeast China as a single population, their genetic diversity and population structure have not been fully understood using molecular markers; in particular, the genetic differentiation of geographical populations of this region has not been well investigated.

The objectives of this work were to determine the extent of genetic variation in the wild soybean in Northeast China, to elucidate the geographical structure and genetic differentiation within and between latitude or longitude populations, and, ultimately, to provide valuable information for the scientific protection and efficient utilization of wild soybean resources in Northeast China.

## 2. Materials and Methods

### 2.1. Plant Material

**Figure 1:**
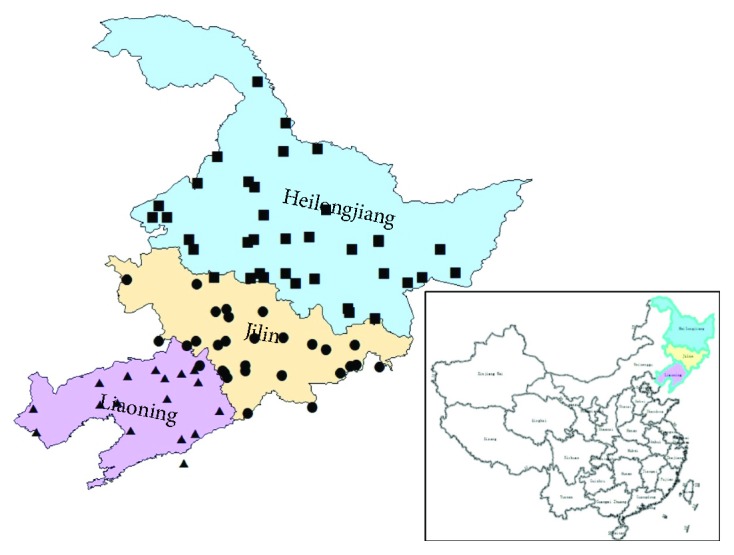
Geographic distribution map of wild soybean accessions in Northeast China. ■, ●, and ▲ represent the wild soybean core collections from Heilongjiang (HLJ), Jilin (JL), and Liaoning (LN), respectively.

All the wild soybean accessions in this study were preserved in the Gene Bank of Jilin Academy of Agricultural Sciences. A total of 205 wild soybean accessions from Northeast China were selected from the core collection established by Zhao et al. [20]. The collection sites of these samples were located within the region of 39–52°N and 119–133°E, covering 84 counties or districts. Nine latitude populations and nine longitude populations were divided based on a degree interval when the sample size was higher than 10; otherwise, the samples were combined into adjacent latitude or longitude population (see Figure 1 and Supplementary Table 1).

### 2.2. SSR Analysis

One single fresh leaf was used to extract genomic DNA for each accession using a modified CTAB method [21]; 43 SSR markers (see Supplementary Table 2), developed from 60 core loci [22] on 20 genetic linkage groups, were used to detect nuclear DNA variation. The primer sequences, with their linkage group locations, are available at https://www.soybase.org/dlpages/#soybasedata.

The 25 *μ*L PCR reaction buffer consisted of 2.5 *μ*L 10x PCR buffer (100 mmol/L), Tris-HCl (pH 8.3), 500 mmol/L KCl, 200 mmol/L MgCl_2_ (0.001% gelatin, 0.1% Np-40), 0.4 *μ*L dNTP (2.5 mmol/L), 2.0 *μ*L each of forward and reverse primers (10 pmol/L), 2.0 *μ*L total DNA (20 ng/*μ*L), 1.0 *μ*L Taq DNA polymerase (2 U/*μ*L), and 15.1 *μ*L ddH_2_O. PCR was performed using the T100™ thermal cycler (Bio-Rad, USA) with the following cycle conditions: an initial denaturing at 95°C for 5 min, followed by 35 cycles of 95°C denaturing for 45 s, 52–57°C annealing for 45 s, 68°C extension for 45 s, and a final extension at 72°C for 10 min.

Amplified products were fractionated by electrophoresis through 6% denaturing polyacrylamide gels and stained with silver staining, which could detect 60 bp to 500 bp fragments and had a high resolution of 2 bp. The size of the stained band was analyzed based on its migration distance relative to the 100 bp DNA ladder (MBI Fermentas) using AlphaView software (version 1.3.0.7).

### 2.3. Data Analysis

The amplification fragments of genomic DNA by each SSR marker were scored based on the migration difference. The data format was converted accordingly in Microsoft Excel. The number of alleles (*N*_a_), Shannon-Weaver index (*I*), expected heterozygosity (*H*_e_), observed heterozygosity (*H*_0_), fixation index (*F*_is_), genetic differentiation coefficient (*F*_st_), genetic identity and genetic distance, molecular variation analysis of variance (AMOVA), Mantel tests, and spatial autocorrelation coefficients were computed by GenAIEx v6.5 [23]. The Shannon-Weaver index (*I*) and expected heterozygosity (*H*_e_) for evaluating the diversity were measured according to the formulas *I* = −1 × sum (*p*_*i*_ × Ln (*p*_*i*_)) and *H*_e_ = 1–sum *p*_*i*_^2^, where *p*_*i*_ is the frequency of the *i*th allele. *H*_0_ is generally lower than *H*_e_ due to inbreeding, and *F*_is_ = (1 − *H*_0_)/*H*_e_. Outcrossing rate (*t*) was calculated using the equation *t* = (1 − *F*_is_)/(1 + *F*_is_) [24]. Based on the matrix of Nei's genetic distance [25], the dendrogram was constructed using NTSYSpc21 [26]. Spatial autocorrelation analysis was performed using “spatial-single pop”; spatial distance was set as 100 km, and the number of permutations and the bootstraps of the selection mode were set as 999 times.

The population genetic structure was predicted by STRUCTURE 2.3.4 [27]. The default *k* value was set to 1 to 12, and ten runs were performed for each value of *k* to test stability of the results. The MCMC (Markov chain Monte Carlo) value was set as 100,000 burn-in with 200,000 iterations. The correct number of genetic clusters was inferred according to a value of Δ*k* (Δ*k* = mean|lnP(*k* + 1) − 2lnP(*k*) + lnP(*k* − 1)|/Sd|lnP(*k*)|) [28].

## 3. Results

### 3.1. SSR Polymorphism and Geographic Variation

Two hundred five representative wild soybean accessions across Northeast China were used in this study. A total of 973 alleles were detected from 43 SSR loci, and the percentage of polymorphic loci was 100%. The number of alleles per SSR marker varied from 13 (Satt309) to 36 (Satt286), with an average of 22.628. The mean Shannon information index (*I*) and expected heterozygosity (*H*_e_) were 2.528 and 0.879, respectively (see Table 1).

**Figure 2:**
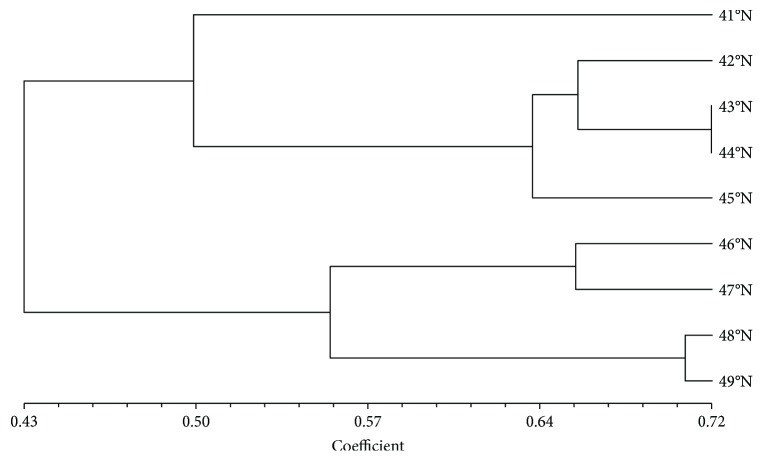
UPGMA dendrogram based on Nei's genetic identity among the latitudes.

At the population level (see Table 2), the Shannon information index (*I*) of nine latitude populations ranged from 1.300 to 2.419, with an average of 1.716; the expected heterozygosity (*H*_e_) ranged from 0.661 to 0.884, with an average of 0.756. The region of 42°N had the highest genetic diversity among all latitudes. The Shannon information index (*I*) of nine longitudes varied from 1.659 to 2.368, averaging 1.919; the expected heterozygosity (*H*_e_) varied from 0.751 to 0.880, averaging 0.805. The region of 124°E had the highest genetic diversity among all longitudes. As shown in Tables 3 and 4, the smaller the latitude difference was, the higher the genetic identity was; however, similar trends were not found in longitude populations. Two major geographical clustering groups (N and S) can be seen in the UPGMA dendrogram. Group N consists of four northern latitude groups (46°N–>49°N), and group S consists of five southern latitude groups (<41°N–45°N) (see Figure 2). The results indicate that the genetic diversity of wild soybean accessions in Northeast China is related to their latitudinal origin.

### 3.2. Population Structure and Genetic Differentiation

The STRUCTURE procedure was run to predict genetic structure for each predefined latitude and longitude population. When *k* was 3, Δ*k* was the highest, which indicated that 3 main clusters had been identified (see Table 5). For the latitude population, 83.8% of individuals from the <41°N region and 50.2% of individuals from the 42°N region were assigned to Cluster1N, most individuals from the 43°N to 44°N region to Cluster2N, and individuals from the 45°N to >49°N regions to Cluster3N. These results were roughly consistent with those of hierarchical cluster analysis (see Figure 2). Some admixtures were found among the 41°N–45°N regions, which probably were important transitional areas. For the longitude population, most of the individuals from the <122°E region were separated from all others and formed a cluster (Cluster1E); three regions of 123°E, 125°E, and 129°E were assigned to Cluster2E, and three regions of 127°E, 128°E, and 130°E to Cluster3L. Admixtures were found widely among 9 predefined longitude populations. The regions of 42°N, 124°E, and 126°E were special, as they were not dominated (<60%) by the three clusters.

The *F*_st_ value was used to evaluate the genetic differentiation of wild soybean populations at the scales of latitude and longitude in Northeast China (see Tables 3 and 4). Pairwise *F*_st_ values for latitude populations ranged from 0.040 to 0.183, and pairwise *F*_st_ values for longitude populations ranged from 0.034 to 0.132. In general, moderate differentiation (0.05 < *F*_st_ < 0.15) [29] was observed between most latitude and longitude populations, and the genetic differentiation among adjacent groups was relatively low. These results were confirmed by AMOVA; most of the genetic variations were found within latitude and longitude populations (see Table 6).

**Figure 3:**
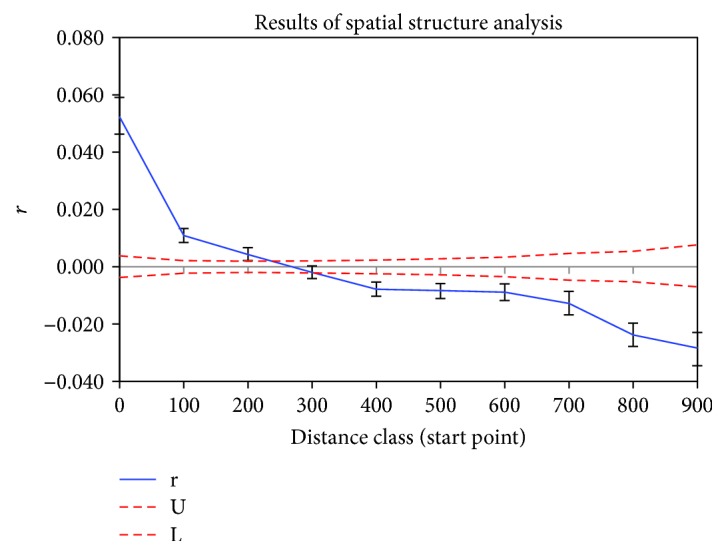
Results of spatial structure analysis. r: solid lines represent spatial autocorrelation coefficients; U and L: dashed lines represent 95% confidence interval.

The Mantel test indicated that there was a positive correlation between geographic and genetic distance (*r* = 0.207, *p* < 0.05), which suggests that geographic distance limits gene flow among populations and influences the genetic structure. For further analyses, spatial autocorrelation analysis was performed by distance classes of 100 km, and a general decline was found in the correlation coefficient (*r*) with distance. The correlation values were negative and significant up to 250 km. This revealed that there is a clinal spatial structure in wild soybeans in Northeast China (*ω* = 119.58, *p* < 0.01) (see Figure 3).

## 4. Discussion

The wild soybean in Northeast China is an important ecotype, and its genetic diversity has been widely studied by using phenotypic traits [7, 30, 31] and molecular markers [17]. The common view has been that the wild soybean in this region possesses a high genetic variation. In this study, the average allele number, Shannon index (*I*), and expected heterozygosity (*H*_e_) were 22.628, 2.528, and 0.879, respectively, which were significantly higher than previously reported results [32–34]. The rich genetic variation could be attributed to the fact that the samples in this study were selected from the core collection, which has been defined as a subset of a crop species preserved with the most abundant repetitiveness [35, 36]. On the other hand, the large sample size and various geographical origins might also result in high diversity; 205 samples used in this study, accounting for 85% of core collections in Northeast China, were selected from 242 core collections developed by Zhao et al. [20]. These accessions may have a continuous distribution in Northeast China (see Supplementary Table 1). Furthermore, 43 SSR primers covering 20 linkage groups might also be important causes for the detection of richer genetic variation [37].

The wild soybean is widely distributed in Northeast Asia. The higher its genetic diversity is, the greater its habitat-expansion capacity and environmental adaptation are [38]. Therefore, it also forms a specific natural distribution pattern [39]. In our study, the results indicate that the region of 42°N and 124°E has the highest level of genetic diversity (see Table 2), and the results roughly agree with those of previous studies based on morphological traits [7]. The results support the view that the genetic diversity of wild soybeans in Northeast China is related to latitude but not to longitude; three evolutionarily significant units were distinguished by latitude, corresponding to regions of <41°N-42°N, 43°N-44°N, and >45°N (see Tables 3 [Table tab4]–5). Moderate differentiation among the latitude populations (the mean *F*_st_ value was 0.088) and longitude populations (the mean *F*_st_ value was 0.058) occurred (see Table 6). This implies that natural selection might be the main cause of genetic structure [8, 29]. Previous studies have revealed that wild soybean genotypes exhibit regional distributions at different geographical scales [15, 40, 41], which are especially associated with latitudinal origin. However, some studies have also reported that the genetic differentiations were associated with longitude origins; for example, Leamy et al.'s results showed that the four genetic groups (Central China, Northern China, Korea, and Japan) differed more in longitude than in latitude [13]. Possible explanations for those results may include small sample size, large geographic span, strait isolation, and diverse ecosystems.

Genetic structure was mainly determined by the breeding system, gene flow, distance isolation, and so on [42]. Wild soybean is a strictly self-pollinating plant, with limited pollen flow. In general, for self-pollination-dominated plants, with an average *G*_st_ = 0.51, the total genetic variation among the populations accounts for more than half of the genetic structure; for out-crossing-dominated species *G*_st_ = 0.10, 90% of genetic variation occurs within populations [43]. In the present study, most of the genetic variation was found between individuals within populations, with less than 10% among populations (see Table 6). This suggests that the genetic differentiation among latitude or longitude populations in Northeast China is similar to that of out-crossing-dominated species [17, 40]. Zhao speculated that this phenomenon could be explained by out-crossing rates and long-distance gene flow [44]. Our results show that the out-crossing rate of Northeast China wild soybeans is only 0.7% (*F*_is_ = 0.987) (see Table 1), which confirms its selfing mating reproductive system and plays an important role in keeping a strong genetic structure.

The Mantel test revealed that there was a positive relationship between geographic and genetic distance (*r* = 0.207, *p* < 0.05), which indicates that geographical isolation has also been an important factor in forming the current genetic structure of wild soybeans in Northeast China. Spatial autocorrelation analysis revealed that the correlation between geographical distance and genetic distance is limited.

The wild soybean in Northeast China has become one of the most severely endangered wild plant species due to the interference of human activities [45]. The genetic diversity of wild soybeans is high, indicating that the wild soybean in this region has great potential for evolution, and in situ conservation is preferable. Although wild soybean accessions ex situ conserved in the National Gene Bank are more substantial than those from other areas, the collection in this area is still very limited, so further investigation and collection works in this region are necessary. According to distribution patterns of the genetic diversity of wild soybeans in Northeast China, the conservation strategy should emphasize individual protection, and protection in areas with high genetic diversity should be prioritized.

## 5. Conclusions

In summary, the genetic diversity and geographic population structure of the wild soybean in Northeast China were fully investigated as a single population, or at different latitude or longitude populations for the first time. The distribution pattern of genetic variation is related to latitude, and the highest level of genetic diversity was found at 42°N, and protection in areas with higher genetic diversity should be prioritized. This study disclosed that natural selection to adapt temperature and photoperiod, selfing mating reproductive system, and distance isolation resulted in the current population structure of wild soybean in Northeast China.

## Figures and Tables

**Table 1:** Genetic diversity of 205 wild soybean accessions by 43 nSSRs.

Number	Primer	LG	*N*_a_	*I*	*H*_0_	*H*_e_	*F*_is_	*t*
1	satt005	Dlb + W	28	2.862	0.025	0.924	0.973	0.014
2	satt022	N	23	2.593	0.010	0.890	0.989	0.006
3	satt099	L	17	2.257	0.020	0.862	0.976	0.012
4	satt112	E	19	2.571	0.010	0.909	0.989	0.006
5	satt146	F	21	2.582	0.034	0.902	0.962	0.019
6	satt168	B2	21	2.743	0.000	0.915	1.000	0.000
7	satt180	C1	18	2.145	0.005	0.813	0.994	0.003
8	satt184	Dla + Q	24	2.658	0.005	0.900	0.995	0.003
9	satt197	B1	31	2.633	0.005	0.876	0.994	0.003
10	satt216	Dlb + W	26	2.295	0.005	0.835	0.994	0.003
11	satt226	D2	22	2.477	0.005	0.888	0.994	0.003
12	satt236	A1	17	2.495	0.015	0.903	0.984	0.008
13	satt239	I	22	2.295	0.005	0.822	0.994	0.003
14	satt242	K	22	2.580	0.005	0.882	0.994	0.003
15	satt243	O	26	2.951	0.015	0.937	0.984	0.008
16	satt267	Dla + Q	18	2.440	0.059	0.890	0.934	0.034
17	satt268	E	18	2.284	0.005	0.859	0.994	0.003
18	satt279	H	28	2.815	0.005	0.916	0.995	0.003
19	satt281	C2	29	2.869	0.005	0.920	0.994	0.003
20	satt286	C2	36	3.124	0.020	0.941	0.979	0.011
21	satt300	A1	20	2.540	0.005	0.903	0.995	0.003
22	satt307	C2	26	2.877	0.061	0.923	0.934	0.034
23	satt308	M	27	2.718	0.015	0.893	0.983	0.009
24	satt309	G	13	1.424	0.005	0.586	0.992	0.004
25	satt334	F	20	2.384	0.010	0.862	0.988	0.006
26	satt345	O	26	2.827	0.005	0.921	0.995	0.003
27	satt346	M	16	2.075	0.005	0.787	0.994	0.003
28	satt352	G	24	2.675	0.010	0.902	0.989	0.006
29	satt373	L	22	2.311	0.005	0.847	0.994	0.003
30	satt386	D2	21	2.489	0.005	0.891	0.994	0.003
31	satt390	A2	25	2.593	0.000	0.894	1.000	0.000
32	satt429	A2	26	2.786	0.005	0.916	0.995	0.003
33	satt431	J	23	2.628	0.005	0.893	0.995	0.003
34	satt434	H	19	2.394	0.000	0.878	1.000	0.000
35	satt453	B1	20	2.301	0.005	0.847	0.994	0.003
36	satt462	L	23	2.618	0.010	0.895	0.989	0.006
37	satt487	O	19	2.279	0.005	0.866	0.994	0.003
38	satt530	N	19	2.330	0.005	0.868	0.994	0.003
39	satt571	B2	21	2.520	0.026	0.897	0.971	0.015
40	satt586	F	33	2.994	0.005	0.932	0.995	0.003
41	satt588	K	26	2.619	0.000	0.896	1.000	0.000
42	satt590	M	22	2.570	0.029	0.899	0.967	0.017
43	satt596	J	16	2.071	0.005	0.835	0.994	0.003
	Mean		22.628	2.528	0.011	0.879	0.987	0.007

LG = linkage group.

**Table 2:** Genetic diversity of different geographical populations in Northeast China.

Longitude Pop.	*I*	*H*_e_	Longitude Pop.	*I*	*H*_e_
<41°N	1.915	0.808	<122°E	1.959	0.820
42°N	2.419	0.884	123°E	1.808	0.789
43°N	2.063	0.816	124°E	2.368	0.880
44°N	1.621	0.751	125°E	2.067	0.833
45°N	1.882	0.790	126°E	2.094	0.827
46°N	1.302	0.661	127°E	1.771	0.780
47°N	1.521	0.733	128°E	1.661	0.769
48°N	1.300	0.662	129°E	1.880	0.797
>49°N	1.419	0.699	>130°E	1.659	0.751
Total	1.716	0.756	Total	1.919	0.805

Pop. = population.

**Table 3:** Nei's genetic distance and genetic differentiation among latitude populations.

Group	<41°N	42°N	43°N	44°N	45°N	46°N	47°N	48°N	>49°N
<41°N	—	0.042	0.079	0.101	0.111	0.183	0.150	0.178	0.162
42°N	0.447	—	0.040	0.061	0.062	0.120	0.091	0.119	0.105
43°N	0.674	0.346	—	0.049	0.066	0.139	0.111	0.140	0.119
44°N	0.790	0.489	0.334	—	0.064	0.156	0.120	0.142	0.126
45°N	0.941	0.499	0.435	0.402	—	0.096	0.080	0.118	0.112
46°N	1.428	0.801	0.818	0.825	0.446	—	0.093	0.155	0.163
47°N	1.441	0.788	0.800	0.784	0.486	0.417	—	0.086	0.098
48°N	1.432	0.858	0.886	0.762	0.615	0.640	0.414	—	0.074
>49°N	1.491	0.896	0.823	0.77	0.674	0.780	0.550	0.349	—

Pop. = population; genetic differentiation coefficient (*F*_st_) (above diagonal); Nei's genetic identity (below diagonal).

**Table 4 tab4:** Nei's genetic distance and genetic differentiation among longitude populations.

Group	<122°E	123°E	124°E	125°E	126°E	127°E	128°E	129°E	>130°E
<122°E	—	0.103	0.049	0.082	0.096	0.124	0.106	0.095	0.132
123°E	0.974	—	0.055	0.052	0.044	0.066	0.075	0.065	0.083
124°E	0.535	0.535	—	0.036	0.039	0.060	0.055	0.049	0.069
125°E	0.818	0.438	0.404	—	0.036	0.052	0.065	0.042	0.076
126°E	0.929	0.345	0.374	0.306	—	0.035	0.035	0.037	0.034
127°E	1.304	0.504	0.563	0.426	0.286	—	0.060	0.061	0.072
128°E	0.980	0.572	0.520	0.537	0.294	0.460	—	0.050	0.056
129°E	0.897	0.500	0.479	0.364	0.298	0.468	0.397	—	0.068
>130°E	1.168	0.545	0.530	0.533	0.227	0.463	0.377	0.443	—

Pop. = population; genetic differentiation coefficient (*F*_st_) (above diagonal); Nei's genetic identity (below diagonal).

**Table 5:** Inferred population structure based on latitude populations and longitude populations.

Pop.	Inferred clusters			Pop.	Inferred clusters		
	Cluster1N	Cluster2N	Cluster3N		Cluster1E	Cluster2E	Cluster3E
<41°N	0.158	0.004	0.838	<122°E	0.891	0.106	0.003
42°N	0.431	0.066	0.502	123°E	0.078	0.690	0.232
43°N	0.916	0.019	0.065	124°E	0.462	0.360	0.179
44°N	0.943	0.054	0.002	125°E	0.122	0.714	0.164
45°N	0.246	0.741	0.013	126°E	0.029	0.433	0.539
46°N	0.034	0.962	0.003	127°E	0.002	0.303	0.695
47°N	0.021	0.977	0.003	128°E	0.053	0.305	0.642
48°N	0.052	0.946	0.002	129°E	0.110	0.614	0.276
>49°N	0.151	0.847	0.002	>130°E	0.003	0.203	0.795

Pop. = population.

**Table 6:** AMOVA analysis of different geographical populations.

Group	Source of variation	SS	MS	Est. Var.	Percentage of variation	*F*_st_	*p*
Latitude	Among pops	723.151	90.394	1.695	9%	0.088	0.001
	Within pops	7058.358	17.602	17.602	91%		
Longitude	Among pops	539.012	67.376	1.111	6%	0.058	0.001
	Within pops	7242.498	18.061	18.061	94%		

Probability, *p* (rand ≥ data), for *F*_st_ is based on standard permutation across the full data set. *F*_st_ = Est. Var. among pops/(Est. Var. among pops + Est. Var. within pops); SS = the sums of squares; MS = the mean sums of squares; Est. Var. = the estimated variance.

## Data Availability

All the wild soybean accessions in this study were preserved in the Gene Bank of Jilin Academy of Agricultural Sciences, and the geographical information for all samples is attached in Supplementary Table 1. The primer sequences with their linkage group locations are available at https://www.soybase.org/dlpages/#soybasedata.

## Supplementary Materials

Supplementary Materials Supplementary Table 1: geographic origin and grouping information of wild soybean core collections in Northeast China. Supplementary Table 2: list of SSR loci.
