# Supplementary material for: The Genetic Diversity and Geographic Differentiation of the Wild Soybean in Northeast China Based on Nuclear Microsatellite Variation
Source: Int J Genomics. 2018 Jun 6;2018:8561458. doi: 10.1155/2018/8561458 (PMC6011050; doi:10.1155/2018/8561458)
Supplement: Supplementary Materials — Supplementary Table 1: geographic origin and grouping information of wild soybean core collections in Northeast China. Supplementary Table 2: list of SSR loci. [file 8561458.f1.docx]

**Supplementary table 1 Geographic origin and grouping information of wild soybean core collections in Northeast China**

| **Geographic origin*** | **Group** | | |  | **Geographic origin *** | **Group** | | |
| --- | --- | --- | --- | --- | --- | --- | --- | --- |
|  | **Province** | **Longitude**** | **Latitude **** |  |  | **Province** | **Longitude**** | **Latitude**** |
| Acheng (1) | HLJ | 126°E | 45°N |  | Fusong (1) | JL | 127°E | 42°N |
| Aihui (4) | HLJ | 127°E | >49°N (50) |  | Fuyu (1) | JL | 124°E | 45°N |
| Baoqing (2) | HLJ | >130°E (132) | 46°N |  | Hailong (4) | JL | 129°E | 42°N |
| Beian (3) | HLJ | 126°E | 48°N |  | Helong (2) | JL | 129°E | 42°N |
| Binxian (1) | HLJ | 127°E | 45°N |  | Huaide (4) | JL | 124°E | 43°N |
| Boli (1) | HLJ | >130°E (130) | 45°N |  | Huichun (2) | JL | >130°E (130) | 42°N |
| Daqing (2) | HLJ | 124°E | 46°N |  | Huinan (5) | JL | 126°E | 42°N |
| Dedu (4) | HLJ | 126°E | 48°N |  | Jian (2) | JL | 126°E | <41°N (41) |
| Dongning (1) | HLJ | >130°E (130) | 44°N |  | Jiaohe (1) | JL | 127°E | 43°N |
| Duerbete (1) | HLJ | 124°E | 46°N |  | Jiutai (3) | JL | 125°E | 44°N |
| Gannan (2) | HLJ | 123°E | 47°N |  | Lishu (1) | JL | 124°E | 43°N |
| Haerbin (2) | HLJ | 126°E | 45°N |  | Liuhe (3) | JL | 125°E | 42°N |
| Hailin (1) | HLJ | 129°E | 44°N |  | Meihekou (1) | JL | 125°E | 42°N |
| Hailun (2) | HLJ | 126°E | 47°N |  | Nongan (1) | JL | 125°E | 44°N |
| Hulin (1) | HLJ | >130°E (132) | 45°N |  | Panshi (2) | JL | 126°E | 42°N |
| Huma (2) | HLJ | 126°E | >49°N (51) |  | Shuangliao (11) | JL | 123°E | 43°N |
| Jiamushi (3) | HLJ | >130°E (130) | 46°N |  | Shuangyang (2) | JL | 125°E | 43°N |
| Jidong (1) | HLJ | 131°E | 45°N |  | Shulan (1) | JL | 126°E | 44°N |
| Jixian (2) | HLJ | >130°E (130) | 46°N |  | Taonan (1) | JL | <122°E (122) | 45°N |
| Longjiang (1) | HLJ | 123°E | 47°N |  | Tumen (1) | JL | 129°E | 42°N |
| Mixian (4) | HLJ | >130°E (131) | 45°N |  | Wangqing (1) | JL | 129°E | 43°N |
| Nehe (1) | HLJ | 124°E | 48°N |  | Yanji (5) | JL | 129°E | 42°N |
| Nenjiang (2) | HLJ | 125°E | >49°N (49) |  | Yaoan (1) | JL | <122°E (122) | 45°N |
| Ningan (1) | HLJ | 129°E | 44°N |  | Yitong (2) | JL | 125°E | 43°N |
| Qingan (2) | HLJ | 127°E | 46°N |  | Yongji (2) | JL | 126°E | 43°N |
| Qinggang (1) | HLJ | 126°E | 46°N |  | Beizhen (1) | LN | <122°E (121) | <41°N (41) |
| Qiqihaer (1) | HLJ | 123°E | 47°N |  | Donggou (1) | LN | 124°E | <41°N (39) |
| Shangzhi (2) | HLJ | 127°E | 45°N |  | Fengcheng (1) | LN | 124°E | <41°N (40) |
| Shuangcheng (17) | HLJ | 126°E | 45°N |  | Fushun (1) | LN | 123°E | <41°N (41) |
| Sunwu (1) | HLJ | 127°E | >49°N (49) |  | Fuxin (1) | LN | <122°E (121) | 42°N |
| Tieli (3) | HLJ | 128°E | 46°N |  | Haicheng (1) | LN | 122°E | <41°N (40) |
| Wangkui (1) | HLJ | 126°E | 46°N |  | Heishan (1) | LN | 122°E | <41°N (41) |
| Xuke (1) | HLJ | 128°E | >49°N (49) |  | Huanren (1) | LN | 125°E | <41°N (41) |
| Yanshou (2) | HLJ | 128°E | 45°N |  | Jianchang (1) | LN | <122°E (119) | <41°N (40) |
| Yichun (1) | HLJ | 128°E | 47°N |  | Jianping（4） | LN | <122°E (119) | <41°N (41) |
| Yilan (2) | HLJ | 129°E | 46°N |  | Kaiyuan（8） | LN | 124°E | 42°N |
| Zhaoyuan (1) | HLJ | 125°E | 45°N |  | Kangping (1) | LN | 123°E | 42°N |
| Antu (2) | JL | 128°E | 43°N |  | Kuandian（2） | LN | 124°E | <41°N (40) |
| Changbai (1) | JL | 128°E | <41°N (41) |  | Liaoyuan（9） | LN | 124°E | 42°N |
| Dehui (6) | JL | 125°E | 44°N |  | Qingyuan（2） | LN | 124°E | 42°N |
| Dongfeng (2) | JL | 125°E | 42°N |  | Tieling（2） | LN | 123°E | 42°N |
| Dongliao (5) | JL | 124°E | 42°N |  | Xifeng (1) | LN | 124°E | 42°N |
| Dunhua (2) | JL | 128°E | 43°N |  | Zhangwu（9） | LN | <122°E (122) | 42°N |

*The number in brackets represented the actual number of samples selected.

**The number in brackets was the actual latitude or longitude of the samples selected.

HLJ, JL and LN represented Heilongjing, Jilin and Liaoning province respectively.

**Supplementary Table 2 List of SSR Loci**

| **Locus(LG)** | **Sense primer (5’-3’)** | **Antisense primer (5’-3’)** | **Core motif** |
| --- | --- | --- | --- |
| Satt300(A1) | GCGCCCACACAACCTTTAATCTT | GCGGCGACTGTTAACGTGTC | (ATT)_19_ |
| Satt236(A1) | GCGTGCTTCAAACCAACAAACAACTTA | GCGGTTTGCAGTACGTACCTAAAATAGA | (ATT)_19_ |
| Satt390(A2) | AGTGGCTGATAAAAAAAATACTCA | ATAATCGCGGCACAATAATTC | (ATT)_17_ |
| Satt429(A2) | GCGACCATCATCTAATCACAATCTACTA | TCCCCATCATTTATCGAAAATAATAATT | (ATT)_25_ |
| Satt197(B1) | CACTGCTTTTTCCCCTCTCT | AAGATACCCCCAACATTATTTGTAA | (ATT)_20_ |
| Satt453(B1) | GCGGAAAAAAAACAATAAACAACA | TAGTGGGGAAGGGAAGTTACC | (ATT)_13_ |
| Satt168(B2) | CGCTTGCCCAAAAATTAATAGTA | CCATTCTCCAACCTCAATCTTATAT | (ATT)_16_ |
| Satt577(B2) | CAAGCTTAAGTCTTGGTCTTCTCT | GGCCTGACCCAAAACTAAGGGAAGTG | (ATT)_12_ |
| Satt180(C1) | TCGCGTTTGTCAGC | TTGATTGAAACCCAACTA | (ATT)_16_ |
| Satt281(C2) | AAGCTCCACATGCAGTTCAAAAC | TGCATGGCACGAGAAAGAAGTA | (ATT)_19_ |
| Satt286(C2) | GCGGCGTTAATTTATGCCGGAAA | GCGTTTGGTCTAGAATAGTTCTCA | (ATT)_17_ |
| Satt307(C2) | GCGCTGGCCTTTAGAAC | GCGTTGTAGGAAATTTGAGTAGTAAG | (ATT)_12_ |
| Satt226(D2) | GCGAAACAACTCACTTAAGCAATACAT | GCGTCCTCCTACCTTTCTTATC | (ATT)_18_ |
| Satt386(D2) | GCGGATGATTTTTATAGAATAGATAAT | CTTCGTTGATACCTCAGTAGAGTACAAA | （ATT）_15_ |
| Satt184(Dla+Q) | GCGCTATGTAGATTATCCAAATTACGC | GCCACTTACTGTTACTCAT | (ATT)_13_ |
| Satt267(Dla+Q) | CCGGTCTGACCTATTCTCAT | CACGGCGTATTTTTATTTTG | (ATT)_16_ |
| Satt005(Dlb+W) | TATCCTAGAGAAGAACTAAAAAA | GTCGATTAGGCTTGAAATA | (ATT)_19_ |
| Satt216(Dlb+W) | TACCCTTAATCACCGGACAA | AGGGAACTAACACATTTAATCATCA | (ATT)_19_ |
| Satt268(E) | TCAGGGGTGGACCTATATAAAATA | CAGTGGTGGCAGATGTAGAA | (ATT)_17_ |
| Sat_112(E) | TGTGACAGTATACCGACATAATA | CTACAAATAACATGAAATATAAGAAATA | (AT)_8_(GA)_18_ |
| Satt146(F) | AAGGGATCCCTCAACTGACTG | GTGGTGGTGGTGAAAACTATTAGAA | (ATT)_17_ |
| Satt334(F) | GCGTTAAGAATGCATTTATGTTTAGTC | GCGAGTTTTTGGTTGGATTGAGTTG | (ATT)_16_ |
| Satt586(F) | GCGGCCTCCAAACTCCAAGTAT | GCGCCCAAATGATTAATCACTCA | (ATT)_19_ |
| Satt309(G) | GCGCCTTCAAATTGGCGTCTT | GCGCCTTAAATAAAACCCGAAACT | (ATT)_13_ |
| Satt352(G) | GCGAATGTATTTTTGTTTCTCCATCAA | TGATAAGCCAAAAAATGGAAGCATAG | (ATT)_18_ |
| Satt279(H) | GCGCAAAAGGACGCCCACCAATAG | GCGGTGATCGGATGTTATAGTTTCAG | (ATT)_28_ |
| Satt434(H) | GCGTTCCGATATACTATATAATCCTAAT | GCGGGGTTAGTCTTTTTATTTAACTTAA | (ATT)_32_ |
| Satt239(I) | GCGCCAAAAAATGAATCACAAT | GCGAACACAATCAACATCCTTGAAC | (ATT)_22_ |
| Satt431(J) | GCGTGGCACCCTTGATAAATAA | GCGCACGAAAGTTTTTCTGTAACA | (ATT)_21_ |
| Satt596(J) | TCC CTTCGTCCACCAAAT | CCGTCGATTCCGTACAA | (ATT)_17_ |
| Satt242(K) | GCGTTGATCAGGTCGATTTTTATTTG | GCGAGTGCCAACTAACTACTTTTATGA | (ATT)_26_ |
| Satt588(K) | GCTGCATATCCACTCTCATTGACT | GAGCCAAAACCAAAGTGAAGAAC | (ATT)_18_ |
| Satt373(L) | TCCGCGAGATAAATTCGTAAAAT | GGCCAGATACCCAAGTTGTACTTGT | (ATT)_21_ |
| Satt462(L) | GCGGTCACGAATACAAGATAAATAATGC | GCGTGCATGTCAGAAAAAATCTCTATAA | (ATT)_20_ |
| Sat_099(L) | GCG AAA ATG GCA GAG ATA A | AATGCTAAAAGAGGAATGAAATAA | (AT)_25_ |
| Satt308(M) | GCGTTAAGGTTGGCAGGGTGGAAGTG | GCGCAGCTTTATACAAAAATCAACAA | (ATT)_21_ |
| Satt346(M) | GGAGGGAGGAAAGTGTTGTGG | GCGCATGCTTTTCATAAGTTT | (ATT)_17_ |
| Satt590(M) | GCG CGC ATT TTT TAA GTT AAT GTT CT | GCGCGAGTTAGCGAATTATTTGTC | (ATT)_26_ |
| Satt022(N) | GGGGGATCTGATTGTATTTTACCT | CGGGTTTCAAAAAACCATCCTTAC | (ATT)17 |
| Satt530(N) | CATGCATATTGACTTCATTATT | CCAAGCGGGTGAAGAGGTTTTT | (ATT)_12_ |
| Satt243(O) | GCGCATTGCACATTAGGTTTTCTGTT | GCGGTAAGATCACGCCATTATTTAAGA | (ATT)_17_ |
| Satt345(O) | CCCCTATTTCAAGAGAATAAGGAA | CCATGCTCTACATCTTCATCATC | (ATT)_27_ |
| Satt487(O) | ATCACGGACCAGTTCATTTGA | TGAACCGCGTATTCTTTTAATCT | (ATT)_22_ |

LG=Linkage group.

The primer sequences with their linkage group locations are available at <https://www.soybase.org/dlpages/#soybasedata>.
